# Applications of Mass Spectrometry in Dentistry

**DOI:** 10.3390/biomedicines11020286

**Published:** 2023-01-19

**Authors:** Meletia Kallianta, Eftychia Pappa, Heleni Vastardis, Christos Rahiotis

**Affiliations:** 1 Department of Operative Dentistry, School of Dentistry, National and Kapodistrian University of Athens, 11527 Athens, Greece; 2 Department of Orthodontics, School of Dentistry, National and Kapodistrian University of Athens, 11527 Athens, Greece

**Keywords:** mass spectrometry, proteomics, salivaomics, ambient ionization mass spectrometry

## Abstract

Mass Spectrometry (MS) is one of the fastest-developing methods in analytical instrumentation. As a highly sensitive, universal detector, it can identify known and unknown compounds, which can indeed be found in a minimal concentration. This review aims to highlight the significant milestones in MS applications in dentistry during recent decades. MS can be applied in three different fields of dentistry: (1) in research of dental materials and chemical agents, (2) in laboratory analysis of biospecimens, and (3) as a real-time diagnostic tool in service of oral surgery and pathology. MS applications on materials and agents may focus on numerous aspects, such as their clinical behavior, possible toxicity, or antimicrobial properties. MS is also a valuable, non-invasive tool for biomarkers’ detection in saliva and has found great application in -omics technologies as it achieves efficient structure-finding in metabolites. As metabolites are located beyond the central dogma, this technique can provide a complete understanding of cellular functions. Thus, it is possible to determine the biological profile in normal and pathological conditions, detect various oral or systematic diseases and conditions, and predict their course. Lastly, some promising advances concerning the surgical approach to potentially oral malignant or malignant disorders exist. This breakthrough method provides a comprehensive approach to dental materials research and biomarker discovery in dental and craniofacial tissues. The current availability of various ‘OMIC’ approaches paves the way for individualized dentistry and provides suggestions for clinical applications in the point-of-care hubs.

## 1. Introduction

**Figure 1:**
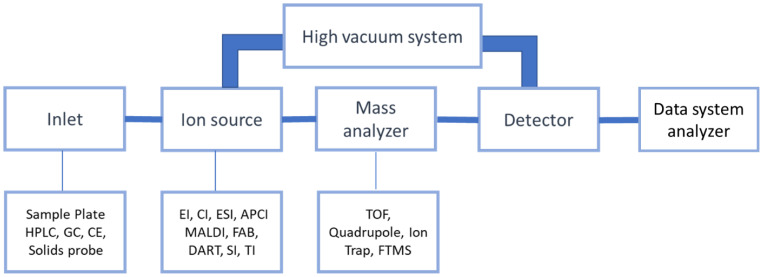
Essential parts of an MS device.

Mass spectrometry (MS) is the analytical technique in which molecules–components of a sample are converted into rapidly moving ions, separated based on their mass and charge ratio (*m*/*z*). A detector captures the dissociated ions and translates them into an electrical signal. The sample is introduced with constant flow, constantly at a gas condition, as it is heated in a high vacuum environment. A typical mass spectrometer device includes an inlet, an ion source, a mass analyzer, a detector, and a data system analyzer, as shown in Figure 1 [1,2]. 

The ionizer adds charges to the sample, and based on the state of the sample, the ionization process may be differentiated. After ionization, the “charged” molecule is directed to the mass analyzer and the detector. “MS” refers to a device with an analyzer and a detector. Thereafter, particular MS technical features signal the monikers, which simultaneously indicate details of the laboratory procedure. Before ionization, the sample composite is typically separated using chromatographic techniques, with few exceptions for other separation methods. In the gas chromatographic technique followed by MS (GC–MS) the mobile phase, as the name indicates, is a gas and usually volatile or lipoid substances [3]. In liquid chromatography followed by MS, respectively LC–MS, the mobile phase is liquid and proteinaceous; this technique can separate organic substances [4]. The CE–MS moniker is used for the case of a capillary electrophoresis liquid separation system [5]. Some MS setups also separate the ions based on their drift time under an applied electrical field before they enter MS. These devices are called Ion Mobility Spectrometry–MS or IMS–MS.

A fundamental principle of the method is that different samples need different ionization sources, analyzers, and detectors. Therefore, each ionization method has unique benefits and drawbacks.

The main ionization techniques in MS include Electron Impact Ionization (EI), Chemical Ionization (CI), Electrospray Ionization (ESI), Atmospheric Pressure Chemical Ionization (APCI), Matrix-Assisted Laser Desorption Ionization (MALDI), Fast Atom Bombardment (FAB), and other sources, such as Direct Analysis in real time (DART), Secondary Ion (SI), and Thermal Ionization (TI) [1,6].

There are also different types of analyzers. A basic technique is governed by the principle that specific electric potential forwards different ions to the detector; hence, the time it takes for each ion to hit the detector is different based on its *m*/*z* value. The “time of flight” is the unit of measurement for this type of mass analyzer, referred to as the TOF analyzer [1].

Another type, quadrupole, uses oscillating electrical fields to stabilize or destabilize the path of ions and allow only the ions of a specific range of *m*/*z* ratio to pass through it. This type of analyzer has variations, such as quadrupole ion trap, linear quadrupole ion trap, or cylindrical ion trap [7]. Fourier Transform Mass Spectrometry (FTMS) detects the mass of the ions by capturing the image created by cyclotron movement under a magnetic field. In Orbitrap, MS ions are trapped under a static electric field [8,9].

A key drawback in the first setup was that each machine could read the sample once. In other words, there was only one mass analyzer for all machines. Tandem MS was created to overcome this restriction, where two or multiple analyzers are included in the setup, and as a result, the sample is read multiple times [10].

1912 was a landmark year when Joseph John Thomson built the first mass spectrometer, and masses of charged atoms were measured. This device used a gas discharge tube to create ions that passed through magnetic and electric fields. The ions were reflected in a parabolic orbit and then traced on a photographic plate. Spectra of O_2_, N_2_, CO, and CO_2_ were obtained for the first time with this device, called “spectrograph”. “Spectrograph,” as a term, had existed in scientific vocabulary since 1884, and represented primordial settings of the mass spectrometer [11].

Nearly a century later, MS has undergone many adjustments and advances and now claims a dominant place among other techniques.

MS can identify known or unknown components of a sample, quantify them, or even interpret their structure. It is characterized by very high sensitivity, reaching up to 10^−18^ M (attomolar range), and high measurement accuracy of the relative molecular masses. Thus, it allows the absolute identification of compounds, even in insufficient quantities. Moreover, as a universal detector, it can reveal the structure of unknown compounds, which has excellent application in -omics technologies. Its reliability, reproducibility, and applicability complement the main features, which establish MS as one of the most advanced technologies in modern biomedical sciences [1].

The current review presents the various applications of mass spectrometry in dental materials research, salivary diagnostics, and real-time detection of oral pathologies.

## 2. MS Applications in Dental Materials

### 2.1. Laboratory Analysis

As mentioned before, MS is a classic technique of analytical chemistry. For this reason, it is a method of choice for the qualitative or quantitative determination of samples’ specific compounds. Such specimens are dental materials, including ceramics, metals, resins, polymers, and biomaterials.

Since the 1990s, there have been studies on the qualification and quantification of leachable residual monomers and additives. These components can elute from various dental composite resins after polymerization. In the study of Stahl et al., published in 1998, the researchers used gas and liquid chromatography/mass spectrometry to identify potential leachable components from four commercial dental composites [12]. Another study aimed to determine an excellent approach to detect minute concentrations of bisphenol-A in dental materials to conclude the potential xenoestrogenisity of eluted resin monomers. Again, GC–MS was the method of choice [13].

In 2017, Nilsen et al. conducted a study analyzing the organic components of resin-modified pulp capping materials. As a result, the authors concluded, using spectroscopic methods, that in these materials, organic compounds elute, are reactive, and are not declared in the safety data sheets of these dental products. The techniques used in this study were Ultra-Performance Liquid Chromatography–Mass Spectrometry (UPLC–MS) and GC–MS [14].

Gas Chromatography MS has also been used to analyze chemical agents with applications in dentistry. In 2021, Sampath et al. investigated the bioactive compounds of guava leaf extract. They reported that these phytochemicals presented antimicrobial, antioxidant, anticancer, and antitumor properties and suggested using this plant in three formulations of toothpaste along with some other ingredients, such as Acacia arabica gum powder, stevia herb powder, and sea salt [15].

In 2021, Zuanazzi et al. conducted a study aiming to evaluate how the composition of the salivary pellicle is differentiated on different titanium modifications. The formed salivary pellicle was analyzed through LC–MS [16].

Chuang et al., in 2017, studied through Time-of-flight Secondary Ion Mass Spectrometry (TOF-SIMS), single or sequential application of silane- and MBP-base primers to zirconia and evaluated the modification of zirconia surface and the effect of these alternations in resin–zirconia adhesion [17]. A similar study was published by Lima et al. in 2019 [18]. In addition, Lapinska et al., in 2019, used the same technique to study the effect that contamination has on the ceramic surface and the effect of contamination removal on the strength of the bond between ceramic and resin [19].

Time-of-flight secondary ion mass spectrometry (TOF-SIMS) has recently been increasingly used in dental materials research. The TOF-SIMS specializes in the chemical qualitative surface analysis of divergent samples covering biological specimens to electronic devices. In dentistry, the method was applied to investigate surface coatings, ceramic surface contamination, regenerative mineralization of biomedical implants, or to examine the zirconium phosphate compound [19]. From what has been mentioned, it is understood that mass spectrometry has wide use in the laboratory analysis of dental materials and their properties. Therefore, all aforementioned studies are listed in Table 1.

In the laboratory applications of MS in dental materials, the technique offers all the advantages of an analytical method. However, its true potential unfolds in the following two sections.

### 2.2. Saliva as a Diagnostic Fluid

Saliva performs multiple functions related to its physical and chemical properties, including antibacterial defense, digestion, lubrication, clearance, rinsing, solubilization, mastication, and swallowing [20]. It is an aqueous solution consisting of 99% water. However, it also includes immunoglobulins, proteins, enzymes, mucins, cells, and electrolytes, such as sodium, potassium, calcium, bicarbonate, and phosphates. Major and minor salivary glands produce saliva, but the final composition of it, referred to as Whole Saliva, is a biofluid that results from salivary glands, gingival crevicular fluid (GCF), expectorated bronchial and nasal secretions, bacteria, viruses, fungi, and their products, desquamated epithelial cells, and other cellular components [21].

Furthermore, blood and its derivatives stand as the primary laboratory diagnostic tool; saliva is a biofluid that gains great diagnostic relevance over time, and it has become a valuable, clinically significant information source. Its composition makes this body fluid an excellent and promising diagnostic agent as it contains many biomarkers, also found in blood, that reflect the state of the oral cavity and teeth. Still, in many cases, it also provides information about systemic diseases. In addition, it offers significant advantages as a diagnostic medium. Its collection is non-invasive, does not require highly trained staff, and is safer to manage, transport, and maintain [22]. The easy, simple, painless, noninvasive sample collection alleviates patients’ discomfort and ensures compliance, especially in vulnerable patient populations, such as neonates and children [23].

The term “Salivaomics” first appeared in science in 2008 and is a subcategory of the more general term “Omics”, which refers to the set of molecules that make up a cell, a tissue, or an entire organism. Salivaomics gathers information from all gene expression levels, including genomics, transcriptomics, proteomics, and metabolomics. This information can be derived from host cells or microbiota, and it can provide an understanding of normal physiology and knowledge about the pathogenesis of various diseases [24]. In this way, all proteomic tools can fill the gaps in the unknown aspects of oral health.

An irreplaceable analytical tool for this rising diagnostic biofluid is mass spectrometry. MS has emerged in recent years as a fundamental technique due to its accuracy and sensitivity in mass measurement and its various techniques can be applied to research biomarkers in saliva [25].

## 3. MS Proteomics

The main techniques of MS used in proteomics are listed below.

### 3.1. 2-DE/MS

**Figure 2:**
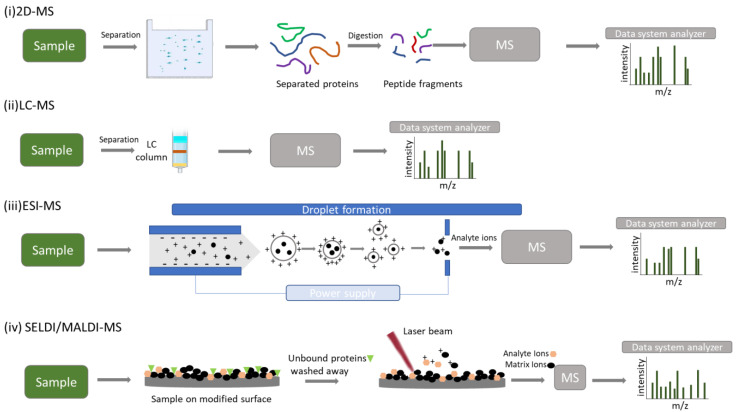
Schematic representation of MS techniques used in proteomics. (**i**) Two-dimensional gel electrophoresis mass spectrometry (2-DE-MS). The sample is separated using gel electrophoresis followed by digestion of proteins and mass spectrometry analysis. (**ii**) Liquid chromatography mass spectrometry (LC-MS). The sample is separated using liquid chromatography followed by ionization and mass spectrometry analysis. (**iii**) Electrospray ionization mass spectrometry (ESI-MS). High voltage is applied to the liquid sample to produce ions (electrospray) and assist the transfer of ions from the solution into the gaseous phase. The resulting charged ions are driven for mass spectrometry analysis. (**iv**) Surface-enhanced laser desorption/ionization/matrix-assisted laser desorption/ionization mass spectrometry (SELDI/MALDI-MS). The sample is applied to a modified surface and contaminants and unbound proteins are washed away (SELDI). A pulsed laser is used to irradiate the sample which is cocrystallized with matrix, resulting in the sample and matrix’s ablation and desorption. Analyte ions are driven for mass spectrometry analysis.

2-DE/MS and bioinformatics tools are the key components of an approach termed ‘‘the classical proteomics methodology’’. This method is considered as the most classical separating technique (Figure 2i). 2-DE, followed by MS, enables the separation of various protein isoforms, yields quantitative results, and provides more information about protein modifications. Its core drawbacks are the labor- and time-intensive approach that is challenging to automate, its repeatability, reproducibility, and low resolution for proteins with molecular weights under 10 kDa. However, as this technique is characterized by simplicity, accessibility, and high throughput, it is expected to be used significantly in the years ahead [26].

### 3.2. LC–MS/MS

When large-scale studies of human saliva arise, gel-based proteomics can be time-consuming and ineffective. Therefore, saliva proteomics has also used liquid-based separation techniques followed by MS (Figure 2ii). A particularly efficient methodology is provided by LC–MS/MS, also known as “bottom-up” proteomics. In addition to the benefits of the high throughput and an extended dynamic range, the sensitivity and selectivity of LC–MS/MS enable quick analysis of low concentrations, even in complex samples. Usually, some sample preparation techniques are needed, which, however, increase sensitivity while also delivering superior precision [24].

### 3.3. ESI–MS

**Figure 3:**
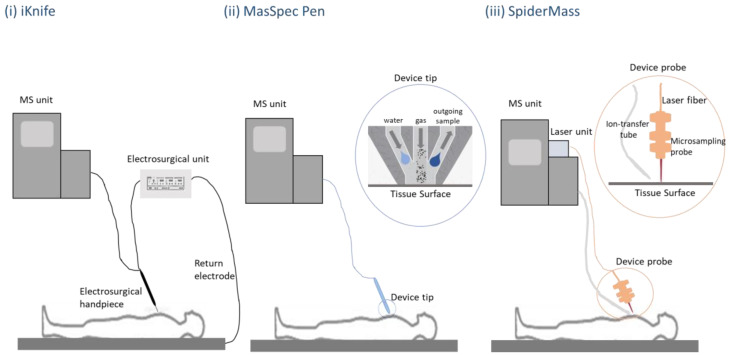
Schematic representation of iKnife, MasSpec Pen, and SpiderMass. (**i**) The Iknife, is known as the intelligent surgical knife that distinguishes normal to malignant tissues. The handpiece delivers aerosols from the electrocauterization to the MS unit for molecular analysis. (**ii**) When the MasSpec Pen is activated, a discrete volume water droplet is exported by the first conduit and interacts with the tissue surface. After 3 s, the gas and vacuum conduits open to transport the droplet with the extracted molecules to the MS unit for molecular analysis. (**iii**) The SpiderMass analysis is performed through a fiber laser microsampling device. The ion transfer tube collects aerosols and transports them to the MS unit.

The ESI technique converts the sample into a fine mist of charged particle droplets under a strong electric field. Its exhaust solvent converts the charged droplets into gas-phase ions. This is one of the essential techniques for analyzing polypeptides, proteins, and oligonucleotides and the main method of coupling LC with MS (Figure 3iii) [27,28].

### 3.4. MALDI-TOF/MS

The great advantage of the Matrix Assisted Laser Desorption/Ionization (MALDI) technique is the ability to measure high molecular weight compounds, such as natural or synthetic polymers, proteins, and peptides. Sample and matrix are mixed, and the resulting mixture is spotted on a MALDI plate where both co-crystallize. A laser beam strikes the plate, and the matrix absorbs energy and transfers it to the analyte molecules. As a result, ions generated by molecules from the matrix and analyte, are ejected into the gaseous phase. MALDI–TOF/MS is considered to be a powerful analytical tool and is widely used for mass spectrometric analysis of large, non-volatile biomolecules [24].

It is also considered ideal for monitoring biomarkers directly from saliva due to its sensitivity, high mass accuracy, high throughput, and quick processing time [29].

This method is characterized by a few drawbacks, including varying sample preparation for various analytes, various discriminatory peaks for comparable samples, and a limited mass window range [1,29].

### 3.5. SELDI-TOF/MS

One of the most promising methods for identifying possible salivary biomarkers for various disorders is SELDI–TOF/MS, which constitutes a MALDI–TOF/MS variation. In this technique, a particular portion of a protein sample is bonded to a chromatographic surface while the remaining portion is washed away (Figure 2iv). The rapid separation, identification, and analysis of proteins and the ability to directly examine undigested native biological samples with high sensitivity are the method’s key benefits, making it particularly suitable for studying low-molecular-weight proteins. However, the primary limitations of this technique are that high molecular weight proteins cannot be analyzed, and its resolution and mass accuracy are inferior to those of MALDI–TOF/MS [30].

All aforementioned techniques are represented in Figure 2.

There are two main strategies through which the proteome analysis is conducted, the bottom-up and the top-down [31]. Through the bottom-up method, MS allows conclusions to be drawn about the proteins in the undigested sample.

It is common practice to identify proteins and determine their sequence and posttranslational modifications using the bottom-up method [32]. Using this method, target proteins are subject to enzymatic, proteolytic digestion from which protein fragments are derived and examined using ESI or MALDI. These mass spectrometry methods enable the gas phase transfer of peptide and protein molecule ions without fragmentation. There are two steps to the ESI– or MALDI–mass spectrometry analysis. The determination of the masses of the intact peptides takes place at the first step, and then the sequence and modifications of these peptide ions are revealed by fragmenting them in the gas phase [31].

The top-down technique determines the protein’s molecular mass when intact protein ions are introduced into the gas phase, fragmented, and examined in a mass spectrometer. This method can thoroughly describe the protein’s fundamental structure, reveal its modifications, and disclose any relationships between these modifications if enough informative fragment ions are observed [33]. In any case, for the analysis of the samples to take place, it is necessary to precede protein purification with techniques such as LC, native and denaturing one-dimensional (1-D) and two-dimensional (2-D) gel electrophoresis, and Western blotting. The most used technique for this process is liquid chromatography, which separates proteins in accord with several properties and is followed by electrophoretic separation [34].

Since the 1980s, mass spectrometry was typically a routine technique for the characterization of small organic compounds [35]. However, macromolecules, such as DNA, proteins, and complex carbohydrates, were proving difficult. The main challenge was giving these big biological molecules enough energy to propel them into the gas phase without destroying them since mass analysis depends on the detection of ionized species in the gas phase. ESI and MALDI are two techniques developed in the late 1980s and made macromolecules available for mass spectrometric examination. Michael Karas and Franz Hillenkamp laid the foundations for Koichi Tanaka who finally developed the MALDI method and was awarded a Nobel Prize. In parallel, John Fenn was also awarded a Nobel Prize for giving “electrospray wings to molecular elephants” [28].

## 4. MS Applications in Salivary Diagnostics

Oral fluids have been significantly studied, and saliva is considered “the mirror of the body” [36]. Therefore, several studies have been conducted on whole-mouth saliva (WMS) and human gingival crevicular fluid (GCF) to evaluate physiological and pathological conditions. The core applications of MS in oral fluids are presented below, and the reports concern either oral or systematic manifestations.

### 4.1. Oral Diseases

#### Caries

Susceptibility to caries results from the imbalance between oral microorganisms and saliva’s protective properties, which, through specific biomolecules, regulate the defense against cariogenic bacteria and modulate oral microbiota. Current research focuses on identifying and quantifying salivary microbes that could be utilized as biomarkers for caries risk assessment and exploring salivary proteins that could be used to predict caries susceptibility [37].

Several studies investigate saliva proteomes to detect caries prevention, diagnosis, and prognosis biomarkers [38]. In 2006, a study was published by Vitorino et al. in which 2-DE, followed by MALDI–TOF MS, was used to investigate the effect of the protein composition of saliva on in vitro formation of the dental pellicle and its possible contribution to caries. Statistical analysis of their results showed a significant correlation between specific molecules and the observed absence of dental caries. In contrast, other molecules correlate with the caries-sustainable group [39].

A study on older adults by Preza et al. in 2009 investigated the secretion of proteins from the parotid gland and suggested that aging tends to change the gland’s function, which may contribute to caries activity. There were also considerable variations in protein patterns among subjects. In this study, elderly with root caries, elderly as controls, and adults without root caries constituted the sample. The detection method of choice was liquid chromatography/tandem mass spectrometry to yield lists of significant proteins in the three groups [40].

Another study conducted on children in 2014, aged from 6 to 8, used electrospray ionization ion-trap tandem mass spectrometry (ESI–MS/MS) to compare the salivary proteome of children with and without dental caries, explore the connection between salivary proteome and dental caries, and advance biomarker investigations of dental caries susceptibility [41].

Pappa et al., in 2021, performed bioinformatics and functional analysis of MS-analyzed proteomic datasets from samples of adolescents with regulated and unregulated type 1 diabetes. They reported the deregulation of significant biological pathways and highlighted specific molecular characteristics in the cariogenic process [42].

### 4.2. Periodontal Disease

Periodontitis is a chronic inflammatory disease characterized by irreversible periodontal attachment loss and alveolar bone destruction. The World Health Organization ranks periodontitis as one of the leading causes of tooth loss [43].

Periodontitis presents a high prevalence and affects the quality of a patient’s life, causing a significant public health problem. So far, the procedure for diagnosing periodontal disease is based on clinical measures of probing pocket depth, bleeding upon probing, plaque index, clinical attachment level, and radiographic examination. However, using the available diagnostic tools, a large-scale screening is difficult to carry out as it is time consuming and requires excellent specialization and technical skills. Consequently, alternative methods should be developed so that a minimally invasive screening for periodontal health can be conducted before an advanced stage of periodontitis is reached, especially in patients with relevant risk factors [44].

The contribution of MS was significant in this case as well. Several studies have used MS in the field of periodontology to analyze pathological protein patterns of gingival inflammation and to differentiate the protein profiles between periodontal and healthy subjects. In 2010, Ngo et al. were the first to use MS for gingival crevicular fluid GCF protein investigation. In the treatment maintenance phase, they identified 33 peptides and 66 proteins in GCF from inflammatory sites in periodontal subjects. All the peptides were discovered to match the MS method’s cleavage products, and 43 of the identified proteins, such as actin and the actin-binding proteins profilin, cofilin, and gelsolin, have not been reported in GCF previously. Specifically, reverse-phase High-Pressure Liquid Chromatography (RP-HPLC) followed by MALDI–TOF/MS has been used for this study as a new method for identifying GCF peptides, and the authors suggested it is a very effective tool in peptide identification compared to previously used techniques [45]. It is a fact that MS techniques have been used since 2000 and 2005 to identify proteins in GCF, such as studies by Kojima et al. and Pisano et al., respectively [46,47].

Three years later, the same authors used the above technique and suggested that GCF mass spectra from periodontal maintenance subjects could be significant indicators for analyzing attachment loss and predicting periodontal disease progression [48].

More recently, in 2020, Antezack et al. published a study in which they examined samples of unstimulated saliva, gingival crevicular fluid, and dental plaque from periodontal and healthy patients and developed, for the first time, diagnostic tests based on their protein profile. These tests showed that MS could be used for classifying these three types of samples according to periodontal status [44].

Furthermore, several studies attempted to identify various biomarkers in the whole saliva by MS techniques and correlated them with different clinical conditions, such as aggressive periodontitis, chronic periodontitis, or periodontitis in obese patients [49,50,51]. These studies aimed to find various combinations of biomarkers that could be used as indicators for every periodontal status, either from a pathogenesis comprehensive, a diagnostic, or a prognostic point of view.

### 4.3. Oral Cancer

Epidemiologically, oral cancer is the sixth-most frequent cancer, and oral squamous cell carcinoma (OSCC) accounts for more than 90% of cases. Because most OSCC patients receive a late diagnosis, the World Health Organization reports that this malignant tumor has a 5-year mortality rate of 45%. Consequently, the most urgent issue for oral cancer is early OSCC diagnosis. In the systematic review by Kaur et al., published in 2018, the authors study salivary biomarkers for cancer and pre-cancer screening. They state that salivary proteins make up the majority of candidate biomarkers of oral cancer. The most used techniques in their investigation were two-dimensional gel electrophoresis and tandem mass spectrometry [52]. In 2020, Chi et al., conducted a study investigating potential biomarkers in paired saliva and plasma samples from oral cancer subjects using targeted MS [53]. Their findings suggest that saliva may be better suited for the protein biomarker-based detection of oral cancer. Furthermore, the recently developed technologies that combine the most beneficial aspects of mass spectrometry and immunoassays can offer a quick and less expensive method of determining the concentrations of particular target proteins, in this case, potential oral cancer biomarkers, in complicated diagnostic media [53].

### 4.4. Oral Lichen Planus

Oral lichen planus is a chronic immune-mediated inflammatory disease with malignant potential. Saliva biomarkers have been investigated, which play a role in inflammation and immune response of OLP. In 2006, Yang et al. conducted a study where two-dimensional electrophoresis and matrix-assisted laser desorption/ionization time-of-flight mass spectrometry were applied to the whole saliva of OLP patients and controls [54]. In 2019, Cruz et al. conducted a metabolic analysis to investigate the molecular mechanisms of OLP’s erosive and reticular forms. The results indicated a different metabolic profile between the two clinical types. Contrary to other studies, this one did not use saliva as a sample but formalin-fixed and paraffin-embedded tissue of erosive and reticular oral lichen planus. The method of choice was GC–MS [55].

### 4.5. Oral Microbiome

The oral cavity is an ideal environment for the growth of microbes. This is because saliva, when produced by its glands, is sterile. However, it is immediately colonized by bacteria found on teeth and soft tissues of the oral cavity [56]. In total, 775 microbial species have been detected in the human aerodigestive tract.

Developing rapid and low-cost methods that are sensitive and reproducible for the screening identification of microorganisms is a desired goal in modern science. Unfortunately, classical identification methods are based on time-consuming and labor-intensive approaches [57].

MALDI–TOF MS is a method characterized by accuracy and speed in which the cells of microorganisms are ionized with short laser pulses and then accelerated by an electric field in a vacuum until they bump into a mass detector where they are identified. Each microorganism has a characteristic spectral profile. Current methodologies include MALDI methods for microorganism identification, but the novel technology of Tandem MS ensures better sensitivity for small intact proteins and records substantially more signals. This promising approach to identifying the oral microbiome could provide valuable information on proteins, toxins, and antibiotic resistance of the specific species present in medical samples without prior knowledge [58]. In 2016, Sampaio-Maia et al. published a fascinating study correlating oral microbiota with oral and systematic diseases. Researchers suggested that the oral microbiome could be a new target for treating oral pathologies and may contribute to the body’s physical state. They report, in detail, that oral microbiota could indicate oral cancer, caries, periodontitis as well as obesity, diabetes, liver diseases, pancreatic cancer, and colon cancer [57]. Other studies have also correlated oral microbiota with the aforementioned medical conditions. Chen et al. published a study in 2020 where they tried to investigate the etiology of caries in children aged from 6 to 8 years and identify specific microorganisms as potential biomarkers using LC-MS/MS [59]. Pei et al. studied oral microbiota of gingival crevicular fluid from subjects with general chronic periodontitis using GC–MS. They concluded that dysbiosis may play a crucial role in the pathogenesis of periodontitis. Acknowledging the ecological profile of this disease could set the conditions for prediction, diagnosis, prognosis, and a personalized treatment plan [27,60]. However, studies correlate changes in the oral microbiome even with conditions such as pediatric obstructive sleep apnea [61]. This implies the high potential of various “OMIC” approaches in oral microbiota. In fact, there is now a mass of information on the oral microbiome composition [37], the salivary proteome, which consists of more than 1000 proteins [62], and the functional profile of the innate and adaptive immune response. In a comprehensive review, Belibasakis et al. presented how the oral microbiome and general oral ecology could be applied to the concept of individualized dentistry, providing suggestions for its practical applications in the clinic [63].

### 4.6. Systematic Diseases

#### 4.6.1. Sjogren’s Syndrome

One medical condition investigated through saliva biomarkers and MS is Sjogren’s syndrome (SS). SS is an autoimmune disease characterized by lymphocytic infiltration of salivary and lacrimal glands. Consequently, patients experience severe dryness of the mouth and eyes. In various studies, proteomic methods were used to identify protein patterns in SS patients. Hu et al., in 2007, aimed to discover biomarkers in the whole saliva that may be used as diagnostic indicators for SS. They concluded that in the whole saliva, molecular signatures indicate a stimulated immune response and damaged glandular cells. Furthermore, they suggested that some proteins, peptides, and mRNA from the whole saliva could be beneficial, once they have been further validated, for the detection of primary SS [64]. MALDI–TOF MS was used in this study, and it became clear that the proteomic analysis of the whole saliva using MS could constitute a novel, valid, and reproducible tool in the study of SS. Several years later, studies, such as Li et al. in 2022, confirmed through MS that saliva metabolic profiling can be used for SS diagnosis and can reveal pathogenetic mechanisms of the syndrome [65].

#### 4.6.2. Diabetes I and II

Another disease that affects the function of salivary glands is Diabetes type 1 and 2. This medical condition has been shown to lead to a decrease in the secretory capacity of the salivary glands and, thus, indirectly affects oral homeostasis. In studies of diabetes salivary proteome through MS, unique proteins have been detected. The reduction or increase in proteins, typically found in the oral cavity, provides information on the evolution of the disease and its dynamics, and allows diabetes screening, detection, and monitoring [66]. For example, Rao et al. in 2009, characterized through liquid chromatography/tandem mass spectrometry, the whole saliva from control and type-2 diabetic subjects and identified 487 unique proteins [67]. Hirtz et al., in 2006, aimed to examine the salivary proteome changes in type 1 diabetes to identify candidate pathology-related indicators. The proteome analysis of saliva samples from healthy individuals and poorly managed type 1 diabetes patients showed a modification of 23 proteins, and the authors concluded diabetes pathogenic mechanisms [68].

In 2012, in Boston, where the Human Proteome Organization (HUPO) meeting occurred, The Human Diabetes Proteome Project (HDPP) was launched in response to the fast development, severity, and consequences of diabetes. Two-dimensional gel electrophoresis (2DE) combined with MS/MS analysis was used to screen the proteome of essential tissues and organs related to diabetes, including the pancreas, skeletal muscle, liver, and adipose tissue, and reference proteome maps were created [69,70,71]. Furthermore, numerous other quantitative proteomic research studies were devised to identify differentially regulated proteins and detect proteome modifications, where Surface-Enhanced Laser Desorption/Ionization-Time-of-Flight mass spectrometry (SELDI–TOF MS) was used [72]. In 2020, a study focused on alterations in post-translational modifications (PTMs) and protein–protein interactions (PPIs) and how these contribute to the development of diabetes. This study concluded that affinity purification mass spectrometry (AP–MS) and several bioinformatic techniques effectively map large-scale PPI networks around the critical participants in glucose homeostasis [73].

Pappa et al., in 2018, analyzed saliva samples by high-resolution mass spectrometry approaches. The samples were collected from children and adolescents with type 1 diabetes and healthy controls. They reported a list of more than 2000 high-confidence protein identifications, constituting a comprehensive characterization of the salivary proteome. Patients with good glycemic regulation and healthy individuals presented similar proteomic profiles. In contrast, more differentially expressed proteins were identified in the saliva of patients with poor glycemic regulation compared to patients with reasonable glycemic control and healthy children. These proteins involved biological processes relevant to diabetic pathologies, such as endothelial damage and inflammation. Moreover, a putative preventive therapeutic approach was identified based on bioinformatic analysis of the deregulated salivary proteins [74].

### 4.7. Cancer

Saliva has been examined as a diagnostic tool for several types of cancer not related to oral manifestations. For example, Breast cancer is one of the most common malignancies worldwide and is the second leading cause of cancer death in women. Therefore, the development of accurate biomarkers for cancer diagnosis is crucial. In 2006, a significant salivary biomarker for breast cancer was discovered by SELDI–TOF MS [75].

Pancreatic cancer has the highest mortality rate of all primary cancers in developed countries. Therefore, it is imperative to find new biomarkers for this type of cancer so that early diagnosis can be made and increase life expectancy. Several studies have been published concerning the investigation of biomarkers of pancreatic cancer from oral fluids. In these studies, different methods of MS have been used, such as Two-Dimensional-Gel-Electrophoresis followed by quantitative Dimethylation–Liquid Chromatography–Tandem Mass Spectrometry and Capillary Electrophoresis–Mass Spectrometry. All studies concluded that salivary biomarkers detected by MS show high rates of sensitivity and specificity in diagnosing pancreatic cancer [76,77].

Another common type of cancer is gastric cancer. Some studies use mass spectrometry as a biomarker detector in this case. For example, in 2009, Wu et al. suggested that a diagnostic approach for distinguishing the protein expression mass spectra of gastric cancer from non-gastric-cancer saliva can be developed based on the modified expression of four proteins [78].

### 4.8. Graft versus Host Disease

It is striking that through MS and salivaomics, indicators related to pathological entities that are not so common can be detected. In particular, in 2012, Bassim et al. investigated Oral Chronic Graft Versus Host Disease (cGVHD) as a weak understanding of its pathogenesis. This study aimed to determine whether Liquid Chromatography–Tandem Mass Spectrometry (LC–MS/MS) can successfully be used to find protein biomarkers of oral cGVHD. As a result, the authors concluded that mass spectrometry could be considered a noninvasive tool for screening, early identification, and monitoring of cGVHD [79].

Table 2 presents the studies mentioned above and their outcomes. It is well-established that mass spectrometry is widely used in diagnostic procedures as an up-and-coming technique.

To sum up, the successful identification of molecular particularities through MS techniques gives a better understanding of the pathogenesis of several diseases, can differentiate the treatment plan and set its course, and impact the patient’s quality of life. In addition, such knowledge can also be helpful in prevention practices.

In dentistry, except various biological samples such as saliva, gingival crevicular fluid (GCF), and microorganisms, proteomic tools can also analyze tissues, such as enamel, dentin, pulp, gums, and mucosa, to identify the protein profile and, hence, differences between normal and pathological conditions [80,81,82].

## 5. MS Applications in Hard and Soft Dental Tissues

MS has been widely used in the investigation of proteins in hard and soft dental tissues and oral fluids and constitutes the most validated technology in studies concerning proteomics.

### 5.1. Dental Hard Tissues

In 2009, Park et al. conducted a study that was the first to provide dentin protein classification in categories such as transport, cellular organization, immune response, signal transduction, and other unknown functions. The researchers used Sodium-Dodecyl-Sulfate-Polyacrylamide Gel Electrophoresis (SDS-electrophoresis) and LC–MS/MS to identify dentin proteins [83].

In 2012, a study was published by Jagr et al. in which 289 proteins were identified in dentin, of which 92 were previously unknown and had not been previously detected in human dentin. In the same study, 9 novel proteins were detected for the first time in an actual human sample and classified mainly as immunoglobulins. The proteins participate in many functions, such as the formation of the extracellular matrix, peptidase activity, immune response, cytoskeleton protein binding, or formation of the cytoskeleton and cell adhesion molecule activity [84]. Two-dimensional gel electrophoresis followed by MS analyzed the extracted proteins.

All this information helps deepen understanding and knowledge of the physiological functions of dental hard tissues.

More studies must deal with their protein profile for cementum and alveolar bone. However, in 2013 Salmon et al. published a proteomic analysis of cementum and alveolar bone in which 235 and 213 proteins were identified, respectively, by LC–MS/MS [85,86].

### 5.2. Acquired Enamel Pellicle

Except for oral fluids, acquired the enamel pellicle (AEP) had also been studied using LC–MS/MS. In 2007, Siqueira et al. identified 130 proteins in the AEP and classified them according to their nature of origin, chemical properties, and biological functions [87]. Another study in 2013 identified 76 proteins in the AEP isolated from deciduous teeth and 38 peptides identified from 10 proteins indicating that the primary AEP proteome composition is unique and different from the permanent AEP proteome. These suggest that oral pathologies linked with this protein structure may demonstrate different trends in their initiation and open up a new diagnostic horizon in pediatric dentistry [88]. Table 3 presents the studies concerning MS applications in hard and soft dental tissues.

### 5.3. Other Applications

The accuracy and sensitivity that MS gives create ideal conditions for many innovative discoveries and applications. One such example is the study of Campanella et al., which was published in 2019 [89]. The authors collected non-stimulated saliva samples from healthy subjects before, during, and after antibiotic treatment over three weeks. Through MS, they detected volatile metabolites for every treatment step, known as metabolic compounds, produced from microbiota susceptible to antibiotics. The authors concluded that this analytical approach could be considered a novel standpoint, as the variance of salivary metabolites can be a unique indirect method for understanding gut microbiota dynamics. In a broader view of the diagnostic potential of mass spectrometry in dentistry, this technique has been used for several objects. In 2016, a study was published in the Journal of Periodontal Research and suggested, using coupled plasma mass spectrometry, that environmental parameters such as smoking may impact the elementary composition of dental calculus. This implies that dental calculus could be considered a noninvasive diagnostic material [90]. In 2014, Warinner et al. used Liquid Chromatography–Tandem Mass Spectrometry to indicate β-lactoglobulin in human dental calculus as a dairy consumption biomarker from the Bronze era to today [91]. Finally, in the study of Buchholz et al. in 2010, the authors present a method, including accelerator mass spectrometry, used to estimate an individual’s age based on the radiocarbon levels in tooth enamel. They claim that when the identity of a deceased person is unclear, knowing the year of birth can be extremely helpful to police investigations [92].

## 6. MS as a Real-Time Detector

Tissue assessment and diagnosis of malignant or potentially malignant disorders by usual histopathologic techniques is time- and labor-intensive. This might cause delays in decisions during diagnostic and therapeutic procedures. Regardless of the surgical intervention, a cancer surgeon must overcome one big obstacle to achieving negative margins to excite a complete tumor. Many techniques have been used for intraoperative diagnoses, such as based on fluorescent probes or Stimulated Raman scattering microscopy [93]. Mass spectrometry (MS) and, specifically, the development of many ambient ionization MS techniques have been applied during the past ten years for quick molecular identification of cancer tissues and have demonstrated remarkable promise for clinical application [94].

Ambient ionization mass spectrometry (AIMS) was first described in the early 2000s. AIMS is a new analytical approach with a great range of applications that covers scientific fields, such as pharmaceuticals, biomedicine, forensic analysis, and cancer pathology. In general terms, AIMS directly analyzes ionized unprocessed or little pretreated samples in their native environments. In traditional MS techniques, a severe limitation is that the sample must be introduced in a vacuum to be analyzed. This innovative technology allows direct, real-time, rapid, high throughput, in situ, and in vivo analysis of gas, liquid, and solid samples under open-air conditions. Since its first report, many techniques and variants have been adapted to serve specific applications. Solvent-based desorption electrospray ionization (DESI) and plasma-based direct analysis in real-time (DART) techniques were crucial in the development of AIMS (P3). DESI is a method where electrosprayed charged microdroplets and ions of the solvent are directed to the surface of the analyzed sample. As a result, microdroplets interact with the surface, and gaseous ions of the sample’s surface material are produced and headed to the setup’s atmospheric inlet. In the DART technique, analysis is based upon atmospheric pressure interactions of electronically or vibronically excited-state atoms and molecules, respectively, with sample and atmospheric gases. Ion–molecule reactions occur between ions generated from the ion source and the sample’s molecules to produce analyte ions [95,96].

Three known systems have been developed for in vivo and real-time analysis exploited AIMS: Iknife, MasSpec Pen, and SpiderMass.

Iknife is an MS method tested as an in vivo intra-surgical diagnostic technique in humans. It was developed in 2009 and is based on rapid evaporative ionization MS (REIMS). The sample to be analyzed is the microparticles produced during the electrosurgical procedure (Figure 3i). The gas-phase ions are then transmitted through a tube, using a Venturi gas jet pump, to the mass spectrometer. This implies that this method is destructive, using the surgical aerosols formed during electrocauterization, and cannot be used as a diagnostic tool before surgical removal. However, it can be seen as a beneficial method as it provides molecular information and can define the range of the sixth tissue so that it is within healthy limits [96,97,98].

MasSpec Pen is a method reported for the first time in 2017. It can and does provide molecular information during surgical procedures without cutting the sub-examination tissue. The array head is a handheld, pen-sized probe with three conduits combined into a small reservoir. The probe comes into contact with the tissue through a water droplet that comes out from the first conduit and causes efficient extraction of biomolecules from the tissue surface. After 3 s, the remaining two conduits with gas and vacuum open, and the sample is directed towards the spectrometer through the vacuum conduit (Figure 3ii). The system recognizes characteristic lipid patterns that are normal or pathological and are found in malignant lesions. This enables an immediate biopsy that will accordingly guide the operation [97,98].

SpiderMass is an in vivo and real-time analysis tool for guided surgery, allowing the definition of resection margins and detecting metastasis microsites. SpiderMass can also have a diagnostic potential as it can be used for ex-vivo real-time applications. It can record, with low damage, biopsy specimens’ molecular profiles and discriminates signals that are specific to normal or tumor tissues. It allows molecular classification, real-time profile acquisition, and grading. SpiderMass is composed of three distinct parts. Firstly, a fibered laser leads to microtissue ablation and particle creation. It is a painless and low-destructive procedure. Next, these particles are aspirated by a transfer tube, through an atmospheric interface, to a mass spectrometer with a modified source that analyzes them by producing ions (Figure 3iii) [99].

Figure 3 represents the three AIMS techniques developed for real-time diagnosis.

All these methods make surgical procedures more efficient, safe, and cost-effective. However, when positive margins are found postoperatively, patients are subjected to additional operative procedures that cause anxiety and discomfort. Moreover, although additional work is required to decrease the size of the mass spectrometer, and sample interpretation still involves skilled and educated staff, these methods broaden our horizons in the management of malignant or premalignant disorders.

## 7. Conclusions

MS was born in the field of physics, and almost a century later it is considered a universal detector with various uses in many scientific areas. In the dentistry, it plays a major role in Salivaomics as it constitutes a powerful proteomic tool selected to identify proteins and determine their primary structure. Further advancements have made it the method of choice in developing new assays, with increased sensitivity, specificity, speed, and high-throughput chemical information above the existing traditional diagnostic techniques. Its use for the laboratory analysis of dental materials is already established. Still, in Salivaomics and Metabolomics, it prevails in biomarker identification, precision medicine, and the development of individualized patient treatment plans. Indeed, saliva became a promising medium for scientific and clinical research for illness, as compared to blood, offering numerous advantages as a diagnostic sample, including non-invasiveness, low cost, and simplicity of use. Therefore, one has driven the development of the other. However, the evolution of MS goes one step further. Large research laboratories have always been the exclusive realm of mass spectrometry but as a result of its miniaturization, in vivo diagnosis in an operating theatre can occur. Indeed, novel technologies in MS instrumentation have produced compatible, automatic, and user-friendly devices as part of routine medical diagnosis. In a few seconds, safe and accurate decision-making for variant medical conditions is feasible. Despite the obstacles that still need to be overcome, MS approaches are expected to lead to the development of precisely personalized dentistry, and have been correctly implemented for these innovations. During recent decades, advances in MS go hand in hand with the revolution of dentistry, paving new pathways in materials innovations, diagnosis, and therapy.

## Figures and Tables

**Table 1:** Use of MS techniques for dental materials laboratory analysis.

Study	Sample	MS Technique	Outcomes	Reference
Stahl et al.	Resin composite	GC/MS, LC/MS	(Co)monomers, different additives, and manufacturing-related impurities were found in all polymerized composite resin specimens. From these, the co-monomer TEGDMA was isolated in concentrations more significant than those found to be harmful in primary human oral fibroblast cells	[12]
Manabe et al.	Resin composite	GC–MS/MS	Bisphenol-A was being released from dental materials, but the leachable amount was less than the reported dose required for xenoestrogenisity in vivo	[13]
Nilsen et al.	Resin-based pulp-capping materials	GC–MS, UPLC–MS	Investigated resin-based pulp-capping materials contained and eluted several reactive, organic substances that were not declared in the safety data sheets of the respective materials	[14]
Sampath et al.	*Psidium guajava*	GC–MS	A polyherbal toothpaste was prepared with guava leaf powder as a significant ingredient and possessed antimicrobial and antioxidant properties	[15]
Zuanazzi et al.	Titanium surfaces and salivary pellicle	nLC–ESI–MS/MS	Despite reported differences in protein composition on titanium surfaces, most proteins were found on all different surfaces, showing a low surface specificity for protein binding in three modified titanium surfaces	[16]
Chuang et al.	zirconia–resin primers adhesion surface	ToF-SIMS	All MDP-treatment groups showed improved shear bond strength (SBS) before thermocycling, while MDP-base primer and MPS followed by MDP retained higher SBS after this	[17]
Lima et al.	zirconia–resin primers adhesion surface	ToF-SIMS	Chemical treatment influenced all surface parameters	[18]
Lapinska et al.	Contaminated with saliva ceramic surface	ToF-SIMS	Cleaning contaminated leucite with orthophosphoric acid or re-etching lithium disilicate with hydrofluoric acid were the most efficient methods for saliva-contaminated ceramic surfaces	[19]

**Table 2:** Use of MS techniques for diagnostic reasons.

Study	Disease Condition	Sample	Proteomic Tool	Outcomes	Reference
Vitorino et al.	Caries	Whole saliva	MALDI–TOF MS	Cystatins, acidic proline-rich proteins (PRPs) and lipocalin-1 are correlated with the absence of dental caries. Amylase, IgA, and lactoferrin levels were found to correlate with the caries-susceptible group.	[39]
Preza et al.	Caries on elderly people	Ductal parotid gland secretion	LC–MS/MS	Aging tends to modify parotid function, which could have an impact on caries activity.	[40]
Yan et al.	Caries in children aged from 6–8	Unstimulated whole saliva	ESI–MS/MS	In contrast to the caries-free group, the total salivary protein was greater in the active-caries group. The initial discovery of distinct proteins (MMP9, MUC7, LTF, CA6, AZU, and cold agglutinin) may serve as a starting point for the study of biomarkers for dental caries susceptibility.	[41]
Pappa et al.	Caries in adolescents with regulated and unregulated type 1 diabetes	Unstimulated whole saliva	LC–MS	A substantial correlation exists between the frequency of caries and unregulated type 1 diabetes in adolescents. The increasing incidence of caries in this group may be explained by the downregulation of the majority of differentially expressed proteins with a protective effect against caries activity.	[42]
Ngo et al.	Periodontitis	Gingival crevicular fluid	MALDI–TOF/TOF MS, nanoLC–ESI–MS/MS	33 peptides and 66 proteins were identified. All the peptides discovered in this study and 43 of the identified proteins had not been reported in GCF before.	[45]
Ngo et al.	Periodontitis	Gingival crevicular fluid	MALDI–TOF MS	The mass spectra of gingival crevicular fluid could be used to predict attachment loss sites.	[48]
Antezack et al.	Periodontitis	Unstimulated saliva, gingival crevicular fluid dental plaque	MALDI–TOF MS	Development of diagnostic tests based on protein profiles of the samples for periodontitis and healthy subjects.	[44]
Kaur et al.	Oral cancer	Whole saliva	MS/MS	Salivary biomarkers could be utilized as a screening technique to increase the accuracy of early detection and diagnosis of oral cancer and pre-cancer conditions.	[52]
Chi et al.	Oral Cancer	Saliva, plasma	LC–MRM–MS	Saliva, unlike blood samples, had more potential for the successful identification of protein biomarkers for the early detection of oral cancer.	[53]
Yang et al.	Oral lichen planus	Whole saliva	MALDI–TOF MS	Palate, lung, and nasal epithelium carcinoma associated protein (PLNEC), and urinary prokallikrein may be two novel biomarkers that are involved in OLP inflammation and immune response.	[54]
Cruz et al.	Oral lichen planus	Formalin-fixed and paraffin-embedded tissue	LC–MS/MS	There are differences between the metabolic profiles of the reticular and erosive forms of oral lichen planus.	[55]
Buszewski et al.	Oral microbiome	-	MALDI–TOF MS	The development of quick, inexpensive, accurate, and reproducible procedures for the screening and identification of microorganisms.	[56]
Chen et al.	Oral microbiome	Supragingival plaque and unstimulated saliva	LC–MS/MS	Some bacteria could be used as potential biomarkers for children with caries.	[59]
Pei et al.	Oral microbiome	Gingival crevicular fluid	LC–MS/MS	Differential microorganisms and metabolites between periodontal and healthy subjects may be used as potential biomarkers.	[60]
Hu et al.	Sjogren’s Syndrome	Whole saliva	MALDI–TOF MS	Whole saliva from patients with primary SS has molecular signals that reflect damaged glandular cells and a stimulated immune system response.	[64]
Li et al.	Sjogren’s Syndrome	Unstimulated whole saliva	UPLC–HRMS	Saliva metabolic profile of pSS is differentiated between the pSS group and the controls. Panels of metabolites may be useful for the diagnosis of pSS.	[65]
Rao et al.	Diabetes type II	Whole saliva	2D-LC–MS/MS	Identification of Salivary Biomarkers of Type-2 Diabetes	[67]
Hirtz et al.	Diabetes type I	Whole saliva	MALDI–TOF MS	Saliva samples from healthy subjects and poorly managed type 1 diabetes patients showed modulation of 23 proteins.	[68]
Pappa et al.	Diabetes type I in children	Whole saliva	LC–MS	Different patterns of protein expression were found in the subjects who had poor glycemic regulation. These proteins participate in biological processes related to the pathophysiology of diabetes.	[74]
Streckfus et al.	Breast cancer	Stimulated whole saliva	SELDI–MS	SELDI mass spectrometry may be a valuable tool in the development of salivary biomarkers.	[75]
Deutsch et al.	Pancreatic cancer	Unstimulated oral fluid	ion-trap MS	A combination of five biomarkers for PC was found. The majority of these proteins have never been found in oral fluids before, despite being known to be relevant to PC or other gastric malignancies.	[76]
Wu et al.	gastric cancer	Whole saliva	MALDI–TOF MS	The differential expression of specific proteins can be used to create a diagnostic model for separating saliva samples from gastric cancer patients and healthy subjects.	[78]
Bassim et al.	Chronic graft-versus-host disease	Unstimulated whole saliva	LC–MS/MS	Mass spectrometry could be used for noninvasive tests for screening, early detection, and monitoring of cGVHD in large patient population.	[79]

**Table 3:** Use of MS techniques in the investigation of hard and soft dental tissue.

Study	Sample	MS Technique	Outcomes	Reference
Jagr et al.	Dentin	LC–MS/MS	289 proteins were identified with high confidence, 90 of which had not been previously detected in human dentin.	[84]
Salmon	Dental Cementum, alveolar bone	LC–MS/MS	The first analysis of the proteomic composition of human DC matrix and comparison to that of the alveolar bone. The discovery of potential biomarkers may result in periodontal regeneration treatments that are more effective and reliable.	[86]
Siquiera et al.	Acquired enamel pellicle	LC–ESI–MS/MS	Identification of proteins in in vivo acquired enamel pellicle.	[87]
Zimmerman et al.	Acquired enamel pellicle on deciduous teeth	LC–ESI–MS/MS	AEP proteome presents a unique composition.	[88]
